# The HIV-1 Vpr R77Q mutant alters host apoptotic gene regulation in CD4+ T cells

**DOI:** 10.3389/fcimb.2026.1830094

**Published:** 2026-06-29

**Authors:** Sidney T. Sithole, Joshua Ramsey, Macey C. Call, Kenneth K. K. Ewool, Brian D. Poole, Brett E. Pickett, Bradford K. Berges

**Affiliations:** Department of Microbiology and Molecular Biology, Brigham YoungUniversity, Provo, UT, United States

**Keywords:** AIDS, apoptosis, HIV, host–virus interaction, RNA sequencing, transcriptomics, Vpr

## Abstract

**Introduction:**

HIV-1 viral protein R (Vpr) is a multifunctional accessory protein that modulates host cellular pathways and contributes to viral pathogenesis. The HIV-1 Vpr R77Q polymorphism is enriched in long-term non-progressors (LTNPs) and has been associated with reduced overall cell death and a shift toward non-inflammatory apoptotic cell death in CD4+ T cells. However, the host mechanisms underlying this phenotype remain poorly understood.

**Methods:**

To define host responses to this variant, RNA sequencing was performed in the CD4+ T-cell line, HUT78, infected with replication-competent HIV-1 NL4–3 wild-type (WT) or the R77Q mutant across a time course of infection (4–72 hpi). Differential gene expression and functional enrichment analysis were complemented by functional assays, including mitochondrial membrane potential assessments, immunoblotting, and pharmacological and genetic modulation of the BCL-2 protein.

**Results:**

Comparison of infected and mock-treated cells revealed robust host transcriptional responses at early time points (4–24 hpi), consistent with known cellular responses to HIV-1 infection. While R77Q and WT infections showed minimal differences in host gene expression early post-infection, at 72 hpi 289 genes were differentially expressed at significant levels. WT infection induced anti-apoptotic programs, including upregulation of *BCL2*, whereas R77Q failed to activate these survival pathways. PPI analysis revealed a highly interconnected BCL-2 family-centered network governing intrinsic apoptosis. Functionally, R77Q infection resulted in significant mitochondrial depolarization, as evidenced by a JC-1 fluorescence shift indicating reduced mitochondrial membrane potential, consistent with activation of intrinsic apoptotic pathways. At the protein level, BCL-2 expression was significantly higher in WT compared to R77Q-infected cells. Modulation of BCL-2 confirmed its functional role: overexpression abrogated R77Q-induced apoptosis, whereas pharmacological inhibition with Venetoclax shifted WT-induced cell death from necrosis towards apoptosis.

**Discussion:**

These findings demonstrate that the R77Q mutant reprograms host apoptotic signaling by impairing BCL-2-mediated survival pathway and promoting mitochondrial dysfunction. This shift toward pro-apoptotic signaling may provide a mechanistic link between HIV-1 Vpr polymorphism and the LTNP phenotype.

## Introduction

Human immunodeficiency virus type 1 (HIV-1) is the main causative agent of acquired immunodeficiency syndrome (AIDS) (Fauci, 1988; Weiss, 1993; Bekker et al., 2023). AIDS occurs when HIV infects CD4+ helper T cells, killing them and thus inhibiting adaptive immune responses, leaving patients highly susceptible to diseases that would otherwise be detected by the immune system (Chaisson et al., 1998; Huang and Crothers, 2009; Okoye and Picker, 2013; Muturi et al., 2023). Chronic immune activation and inflammation is a hallmark of AIDS development (Dalgleish and O’Byrne, 2002; Hunt, 2012; Deeks et al., 2013; Lv et al., 2021), and a variety of factors are thought to contribute to this activation (Appay and Sauce, 2008; Lv et al., 2021; Mazzuti et al., 2023). Some patients develop AIDS slowly, or not at all, and are commonly referred to as long-term non-progressors (LTNP) (Capa et al., 2022; Love et al., 2022). While host genetic factors contribute to the development of the LTNP phenotype (Telenti and Bleiber, 2006; Casado et al., 2010), substantial evidence exists to support HIV-1 genetics playing a significant role (Emiliani et al., 1996; Zhao et al., 2002; Lum et al., 2003; Andersen and Planelles, 2005; Wang and Su, 2019; Wang et al., 2024).

The viral protein R (*vpr*) gene in HIV-1 is one such gene where polymorphisms have been found to be associated with the rate of AIDS development (Andersen and Planelles, 2005; Langford et al., 2007; Soares et al., 2016). The R77Q mutation was identified in 2003 as a nonsynonymous single nucleotide polymorphism (SNP) that was detected at significantly higher prevalence in LTNP patients (Lum et al., 2003; Rodés et al., 2004). However, the mechanism(s) by which this mutation could cause LTNP are not well understood (Wang and Su, 2019).

Our group previously reported that the R77Q mutation is associated with higher levels of apoptotic cell death in multiple CD4+ T cell lines as well as in cord blood-derived primary CD4+ T cells (Solis-Leal et al., 2024). In contrast, the wild-type NL4–3 strain killed cells through a mechanism involving membrane permeability, which is indicative of necrosis (Golstein and Kroemer, 2007; Vanlangenakker et al., 2008). Furthermore, infected cells showed significantly higher levels of G_2_ cell cycle arrest and lower levels of pro-inflammatory cytokine production (TNF and IL-6), as compared to the wild-type strain. Reduced levels of pro-inflammatory cytokines are a potential link between the R77Q mutant and slower AIDS progression (Thea et al., 1996; de Medeiros et al., 2016), since strong inflammatory signals may be connected to increased chronic immune activation (13, 33–35). However, that study did not identify the signals that contribute to the apoptotic and G_2_ arrest phenotypes (Solis-Leal et al., 2024).

In this study, we performed a bulk transcriptomic analysis (Illumina RNA-Seq) of three populations of cells: a) infection with wild-type strain NL4-3, b) infection with the R77Q mutant (a single point mutation difference as compared to NL4-3, which only affects the *vpr*), and c) mock-infected cells. The HUT78 cell line was used, which was the main cell type used in our previous report (Solis-Leal et al., 2024). Multiple time points were analyzed; however, significant differences in the R77Q vs. WT comparison were only noted at 72 hours post-infection (hpi), which became the focus of subsequent analysis. We performed gene ontology (GO) term analysis and Kyoto Encyclopedia of Genes and Genomes (KEGG) analyses to identify genes and cellular pathways, respectively, that differed in the various samples. We found 289 genes to be significantly differentially expressed when comparing R77Q (case) to WT HIV-1(control). Because of our previous findings related to the induction of apoptosis, we focused on analysis of genes and pathways related to this process. We found that the *BCL2* gene, a key inhibitor of intrinsic apoptosis (De Rossi et al., 1994; Kaloni et al., 2023), was upregulated during infection with WT virus, whereas R77Q failed to induce *BCL2* expression above control levels. Bim, which normally inhibits *BCL2* (O’Connor, 1998; Chen, 2002; Mühleisen et al., 2014), had gene expression significantly upregulated by R77Q as compared to WT virus.

Furthermore, to complement these transcriptomic findings, we performed functional and protein-level analysis to assess the biological consequences of these regulatory differences. These included evaluation of ΔΨm, immunoblot-based quantification of BCL-2 expression, and genetic and pharmacological modulation of BCL-2 activity. Together, these approaches provide an integrated view of how the R77Q mutant reshapes host apoptotic signaling.

These findings shed insight into how the R77Q mutant could trigger a host response characterized by non-inflammatory apoptotic cell death, in contrast to the WT virus, and may help to explain its association with the LTNP phenotype (Burdo et al., 2023).

## Materials and methods

### Cell lines and cell culture

HEK293T cells were maintained at 37 °C, 5% CO_2_ in DMEM (Sigma-Aldrich, USA), supplemented with 10% fetal bovine serum (Sigma-Aldrich, USA) and 1% penicillin/streptomycin (Sigma-Aldrich, USA). Ghost R3/X4/R5 cells were maintained at 37 °C, 5% CO_2_ in DMEM, supplemented with 10% FBS, 1% P/S, 500 µg/mL G418, 100 µg/mL hygromycin, and 1 µg/mL puromycin. Ghost cells were obtained through the NIH HIV Reagent Program, Division of AIDS, NIAID, NIH: GHOST (3) CCR3+ CXCR4+ CCR5+ Cells from Drs. Vineet N. Kewal Ramani and Dan R. Littman (cat# 3934). HUT78 cells were maintained at 37 °C, 5% CO_2_ in RPMI (Sigma-Aldrich, USA), supplemented with 10% FBS and 1% penicillin/streptomycin.

#### Plasmids

HIV-1 pNL4–3 was obtained through the NIH HIV Reagent Program, Division of AIDS, NIAID, NIH: Human Immunodeficiency Virus 1 (HIV-1), Strain NL4–3 Infectious Molecular Clone (pNL4-3), ARP-114, contributed by Dr. M. Martin. The HIV-1 NL4–3 R77Q Vpr mutant was a gift from Dr. Velpandi Ayyavoo (University of Pittsburgh). The *BCL2* overexpression plasmid, pcDNA3-Bcl-2, encoding human BCL-2 was obtained from Addgene (Human Bcl-2/pcDNA3, Addgene cat#19279).

### Virus production and titration

Virus stocks were generated by transfection of HEK293T cells using the calcium phosphate method. Supernatants were harvested 48 hours post-transfection. Viral titers were quantified in Ghost R3/X4/R5 cells following the assay protocol provided by the NIH HIV Reagent Program (Rockville, MD, USA).

### HIV-1 infection assays

Infections were performed in 1 × 10^5^ HUT78 cells at an MOI of 0.3 in the presence of 10 µg/mL polybrene. Mock-infected cells were treated in the same concentration of polybrene and an equivalent volume of virus-free supernatant. All conditions were performed in triplicate.

### RNA extraction and sequencing

Total RNA was extracted from each condition at each time point using the Direct-zol™ RNA MicroPrep w/TriReagent (Zymo Research, USA) extraction kit according to the manufacturer’s instructions. RNA purity and concentration were initially measured using the Thermo Scientific NanoDrop before being shipped to LC Sciences, Houston, TX, for poly-dT based mRNA library preparation and sequencing. RNA integrity was then assessed using the Agilent Technologies 2100 Bioanalyzer (High Sensitivity DNA Chip). All samples demonstrated high RNA integrity, with RNA Integrity Numbers (RIN) ≥ 8.6 and the majority exceeding 9.0, indicating suitability for downstream sequencing analysis. Sequencing was performed on a Illumina NovaSeq 6000 following their internal protocol and the generated FASTQ files were shared via a LC Sciences server. The sequencing reads and processed data obtained during this study have been submitted to the Gene Expression Omnibus (GEO) database, under accession number GSE319139.

### ARMOR and quality control

**Figure 1:**
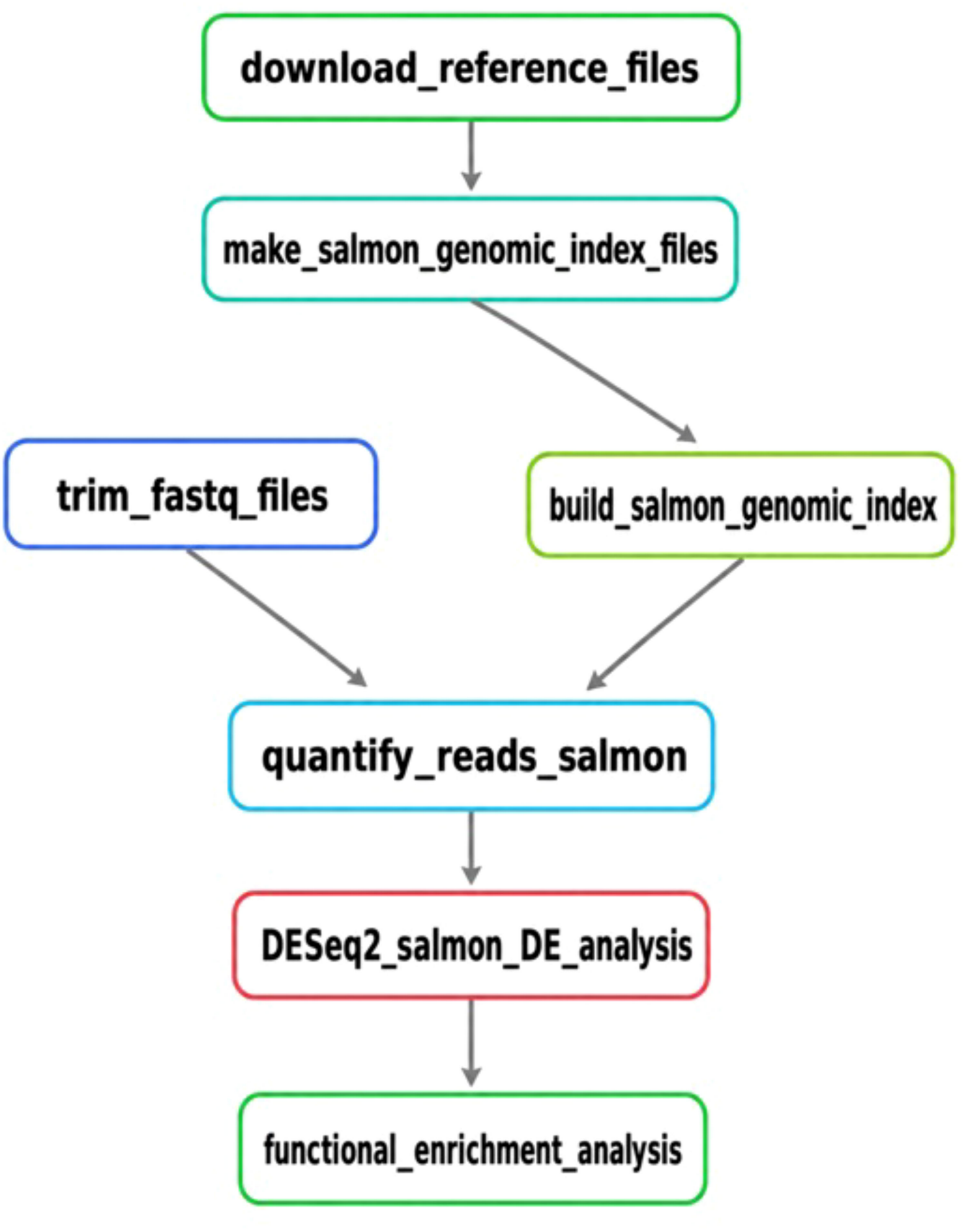
RNA seq data processing flow. Downloaded FASTQ files were processed using the Automated Reproducible Modular workflow for RNA-seq data (ARMOR, v1.5.10). The pipeline included read quality assessment (FastQC v0.11.9), adapter and low-quality base trimming (TrimGalore! v0.6.6), and transcript-level alignment and quantification against the human transcriptome (Salmon v1.4.0). Differential expression (DE) analysis was carried out using DESeq2 package v1.50.2.

Downloaded FASTQ files were run through an existing Automated Reproducible Modular workflow for processing and differential analysis of RNA-seq data (ARMOR) v1.5.10 pipeline as previously described (Orjuela et al., 2019; Wilkins and Pickett, 2025). The ARMOR workflow performed quality control on the sequencing reads (FastQC v0.11.9), trimming (TrimGalore! v0.6.6) of adapters and poor-quality regions and mapping (Salmon v1.4.0) and quantifying sequencing reads to the human transcriptome. This workflow was performed on the MaryLou supercomputer at Brigham Young University, Provo, Utah, USA, using default parameters. The data processing steps are outlined in **Figure 1** as a summary.

### DEGs and functional enrichment analysis

Differential expression (DE) analysis was carried out using DESeq2 package v1.50.2 (Love et al., 2014) which employs a negative binomial distribution model for determining statistical significance. Raw gene-level counts were imported, filtered to remove low-abundance features, and normalized using the trimmed mean of M-values (TMM) method (Robinson and Oshlack, 2010; Smid et al., 2018). Genes with a false discovery rate (FDR) adjusted *p*-value below 0.05 were considered significant.

Differential gene expression was followed by functional enrichment analysis using clusterProfiler v4.18.4 and gprofiler2 v0.2.4. GO term relationships and gene–term connections were visualized using enrichplot v1.30.3, which maps significant DEGs to hierarchical GO structures and highlights shared gene membership across terms (Wu et al., 2021; Sumner, 2024). To evaluate apoptosis-related responses, we filtered enriched GO biological process terms for keywords associated with apoptotic regulation (“apoptosis,” “programmed cell death,” and “cell death regulation”). Over-representation testing was conducted using a hypergeometric distribution and the Benjamini–Hochberg multiple hypothesis testing correction procedure, and pathway-level visualizations were generated with Pathview v1.41.1 (Luo and Brouwer, 2013).

### STRING protein-protein interaction

Predicted protein-protein interaction networks were generated using Search Tool for the Retrieval of Interacting Genes/Proteins (STRING) (v12.0; https://string-db.org) by submitting the list of significantly differentially expressed genes from the R77Q vs. WT comparison at 72 hpi. Network reconstruction was performed using the default settings, including the full STRING evidence channels (Timalsina et al., 2014). The resulting network was exported in both image and tabular formats, the latter containing interaction edges with associated confidence scores. Network visualization and refinement were carried out in the STRING interface, with node size scaled according to degree centrality and edge thickness mapped to interaction confidence to improve interpretability and figure quality.

### Apoptosis analysis by flow cytometry

HUT78 cells were infected as described above and harvested 72 hpi for flow cytometric analysis. Cells were washed with serum-free PBS and pelleted at 500× g for 5 min. Annexin V and Fixable Viability Dye (FVD) (Thermo Fisher Scientific, MA, USA) staining was performed following a method previously described (Solis-Leal et al., 2024). Data were acquired on a Beckman Coulter Cytoflex S Flow Cytometer (CA, USA) and analyzed using FlowJo software (version 10.10.0) and GraphPad Prism (version 11.0.0.84).

### Immunoblotting

Infections were performed as previously described. Cells were harvested 72 hpi, washed in ice-cold PBS and lysed in 1x RIPA buffer. Protein quantification was done using the Pierce BCA protein assay kit (Thermo Fisher Scientific, MA, USA). Equal amounts of protein per sample were boiled with 2x SDS sample buffer (125mM Tris-HCl, 4% SDS, 20% glycerol, 0.01% bromophenol blue and 10% 2-mercaptoethanol) for 5min. Samples were then run through a 12% SDS polyacrylamide gel using standard conditions and transferred onto a nitrocellulose membrane. The membrane was blocked with PBS containing 1% fish gelatin for 1hr at 25°C. BCL-2 was probed using mouse anti-hu BCL-2 primary antibody (1:500) (Thermo Fischer Scientific) overnight at 4 °C. Membranes were then washed and incubated in 10ml of LI-COR goat anti-mouse secondary antibody (1:2000) (IRDye 680CW) for 1hr at room temperature. The proteins were visualized using the LI-COR odyssey CLx-1893 imaging system. The membrane was stripped using stripping buffer (Santa Cruz Biotechnology, sc-281698) for 30 min at 25°C and re-blocked in 50 ml 1% fish gelatin for 1hr. GAPDH was then probed with rabbit-anti-GAPDH primary antibody (1:1000) (Cell Signaling Technologies,#2118) at 4 °C overnight. Membrane was washed and incubated with LI-COR goat anti-rabbit secondary antibody (1:2000) (IRdye 800CW). A quantitative analysis of the fluorescent bands on the membrane was performed using ImageJ software (version 1.54g, NIH, Bethesda, MD, USA) and normalized to a GAPDH loading control.

### Statistical analysis

Differential gene expression was evaluated using standard linear modeling with false-discovery rate (FDR) p-adjusted correction (Benjamini–Hochberg). Genes with a FDR < 0.05 were considered significant. For flow cytometry and immunoblotting experiments, data from biological triplicates were analyzed using a one-tailed Student’s *t*-test, as a directional change in response to infection was hypothesized *a priori*. Statistical analyses were performed using GraphPad Prism (version 11.0.0.84), and significance was defined as *p* ≤ 0.05.

### Overexpression of BCL-2

The pcDNA3-Bcl-2 plasmid and the corresponding empty pcDNA3 vector were transfected into HUT78 cells by electroporation using a Lonza 4D-Nucleofector System (Lonza, Basel, Switzerland). In brief, 2 x 10^6^ cells were resuspended with 2µg plasmid DNA in 100µl of electroporation buffer (Cell Line Nucleofector^®^ Kit R, Lonza, Basel, Switzerland). The cell-DNA suspension was transferred into sterile electroporation cuvettes and subjected to the appropriate Nucleofector program. Immediately following electroporation, cells were incubated at 37°C, 5% CO_2_ in pre-warmed recovery media. At 12 hours post transfection, cells were washed in RPMI-1640 and maintained under standard culture conditions. Stable transfectants were selected by gradual addition of G418 to a final concentration of 100 µg/ml.

### Mitochondrial membrane potential analysis

Mitochondrial membrane potential (ΔΨm) was assessed by flow cytometry using the MitoProbe™ JC-1 assay kit (Thermo Fisher Scientific, MA, USA). JC-1 is a cationic dye that accumulates in mitochondria in a membrane potential-dependent manner, exhibiting green fluorescence in its monomeric form (low ΔΨm) and red fluorescence upon aggregation in polarized mitochondria (high ΔΨm). In brief, HUT78 cells were infected as previously described. At 72 hpi, cells were harvested, adjusted to 1.0 x 10^6^ cells/mL, and incubated with JC-1 at 37°C for 15 minutes according to the manufacturer’s instructions. Following incubation, cells were washed and resuspended in assay buffer. Samples were acquired on a Beckman Coulter Cytoflex S Flow Cytometer (CA, USA) and data were analyzed using CytExpert software (version 2.6).

## Results

### HIV-1 Vpr R77Q- vs. WT-infected cells show distinct and divergent transcriptomic signatures at 72 hpi

To investigate the mechanism underlying HIV-1 Vpr R77Q–induced apoptosis, we analyzed host transcriptomic changes in the human CD4+ T cell line HUT78. We infected cells at a multiplicity of infection (MOI) of 0.3 with replication-competent HIV-1 NL4–3 wild-type (WT) virus or the R77Q mutant in the presence of 10 µg/mL polybrene. Although viral replication kinetics were not directly measured in the present study, our previous report demonstrated no significant differences in replication efficiency between these two strains under comparable conditions at time points (Solis-Leal et al., 2024). We treated mock-infected cells with an equivalent concentration of polybrene and an equal volume of virus-free supernatant. All experimental conditions were performed in triplicate.

We then harvested total RNA at 4, 8, 12, 24, and 72 hours post-infection (hpi). RNA quality assessment demonstrated high integrity across all samples, with RNA Integrity Numbers (RIN) exceeding 9.0 for most samples and a minimum RIN of 8.6 when assessed by LC Sciences, a CRO contracted for sequencing (data not shown). We sequenced sample libraries on an Illumina NovaSeq 6000 platform, yielding an average of 21.5 million reads per sample and approximately 64.5 million reads per condition following quality trimming using FastQC.

**Figure 2:**
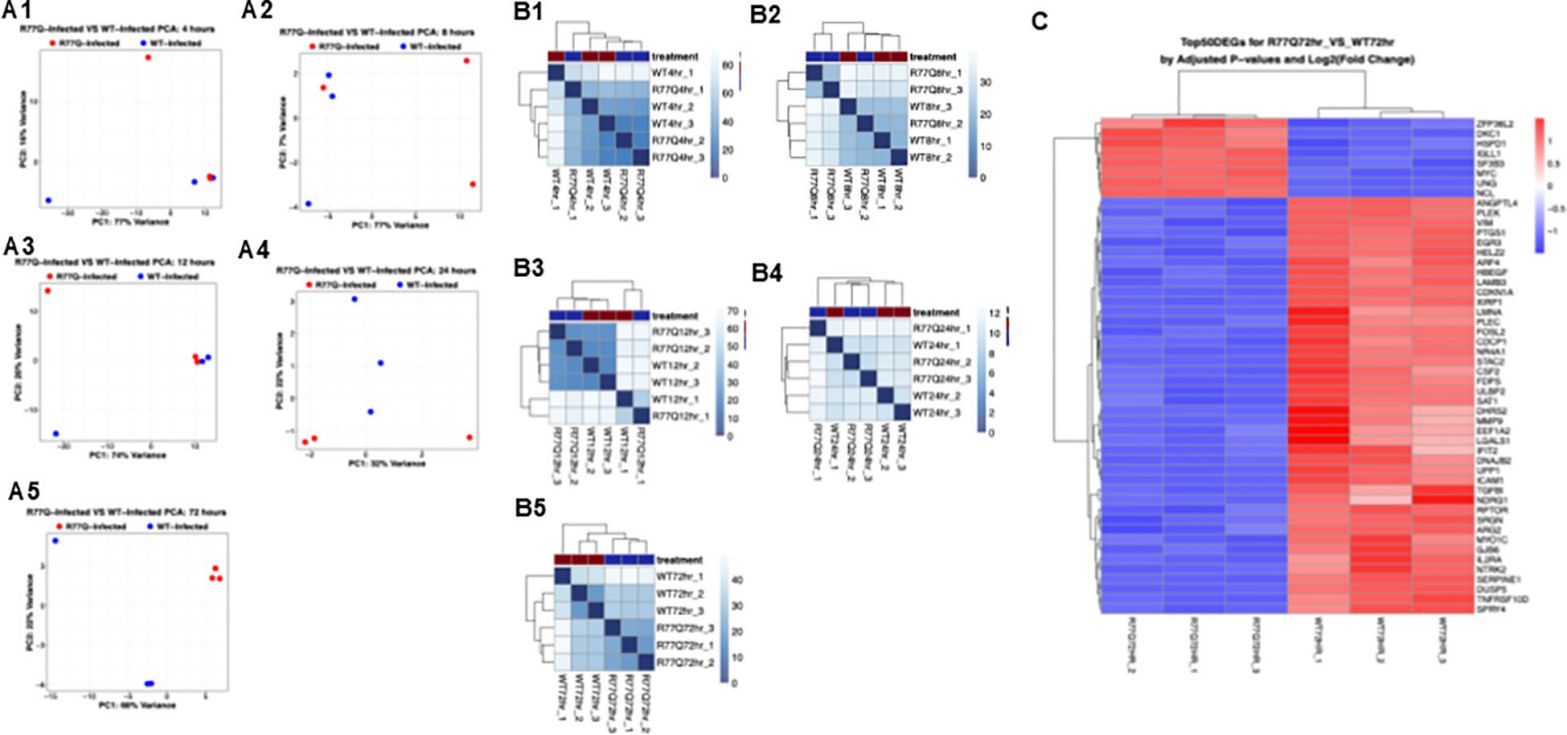
Principal component analysis (PCA) and heatmap analysis show distinct transcriptomic profiles at 72 hpi. **(A1–A5)** PCA at 4, 8, 12, and 24 hpi, respectively, show no clear separation between conditions. **(A5)** By 72 hpi, samples cluster distinctly, with the first and second principal components accounting for 68% (PC1) and 22% (PC2) of the variance. **(B1–B5)** Sample correlation plots at earlier time points show incomplete separation, whereas a clear pattern emerges at 72 hpi **(B5)**. **(C)** Heatmap of the top 50 significant DEGs at 72hpi scaled by *z-score* demonstrates strong within-group correlation and distinct clustering across conditions.

To determine differential gene expression due to Vpr sequence variation, we compared transcriptomic profiles from R77Q- and WT-infected cells across multiple time points (4, 8, 12, 24, and 72 hpi). Principal component analysis (PCA) revealed no clear separation between R77Q vs. WT infected cells at 4, 8, 12, or 24 hpi (**Figures 2A1–A5**), which suggests minimal overall differences across samples. In contrast, robust segregation of R77Q and WT-infected samples was observed at 72 hpi, with principal component 1 and 2 accounting for 90% of the total variance (**Figure 2A5**), indicating substantial transcriptomic divergence at this time point.

Sample correlation analysis further supported these findings. Early time points showed limited correlation among replicates (**Figures 2B1–B4**), whereas samples at 72 hpi displayed strong within-group correlation and clear separation between conditions (**Figure 2B5**). Consistent with the PCA and sample distribution analysis, heatmap visualization of the top 50 most significant genes revealed tight clustering of biological replicates within each condition, with highly similar expression patterns indicative of low intra-condition variability. In contrast, samples from different conditions segregated into distinct clusters and exhibited contrasting expression patterns across gene blocks, consistent with condition-specific transcriptional signatures (**Figure 2C**). Based on quality control and variance assessments, all subsequent differential expression and pathway analyses for the R77Q vs. WT comparison were performed using data from the 72 hpi time point.

### Differentially expressed genes in R77Q vs. WT infected cells

**Figure 3:**
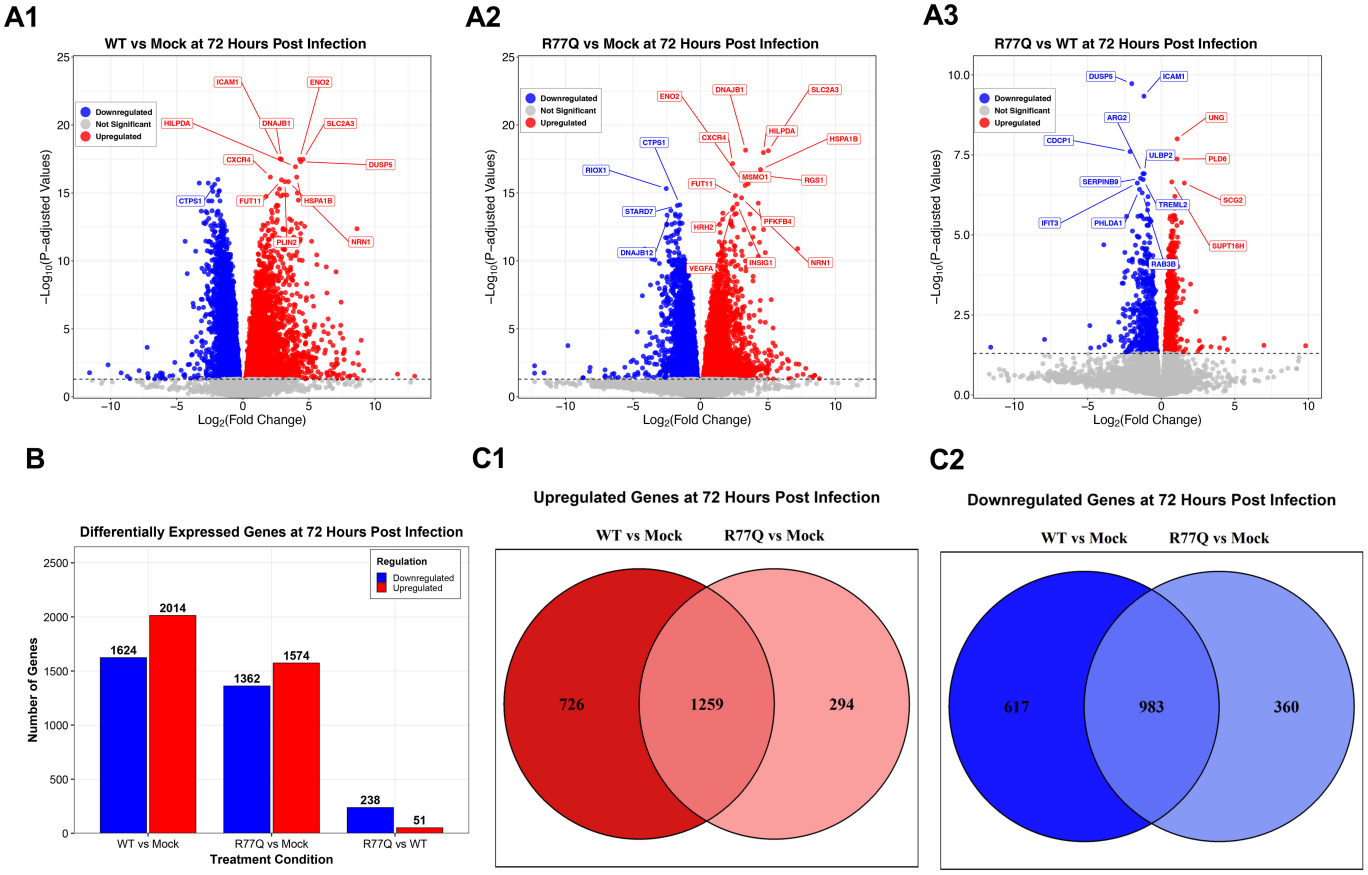
Differential gene expression (DGE) analysis at 72 hpi. A total of 289 differentially expressed genes (DEGs) were observed in R77Q vs. WT-infected cells 72hpi. **(A1–A3)** Volcano plots of WT vs. mock, R77Q vs. mock, and R77Q vs. WT, respectively. Red and blue points represent upregulated and downregulated DEGs respectively while grey dots represent non-significant genes (FDR <0.05) **(B)** Bar graph summarizing the number of DEGs for each comparison at 72 hpi. **(C1–C2)** Venn diagrams showing the overlap of DEGs, separated into downregulated **(C1)** and upregulated **(C2)** sets for each condition.

We determined differentially expressed genes (DEGs) by using a false discovery rate (FDR) adjusted *p*-value threshold below 0.05. Despite differing by a single nucleotide and amino acid, with genome similarity of about 99.99%, WT and R77Q induced 289 DEGs in direct comparison when applying a filtering threshold of |log_2_fold change (logFC)| ≥ 1 (i.e. logFC ≤ -1 or ≥ 1). Relative to mock infection, WT infection resulted in 2014 upregulated and 1624 downregulated genes (**Figure 3A1**), whereas R77Q infection led to 1574 upregulated and 1362 downregulated genes (**Figure 3A2**). Direct comparison of R77Q vs. WT revealed 238 upregulated and 51 downregulated genes (**Figure 3A3**).

We observed substantial overlap when comparing transcriptional differences between cells infected with WT and R77Q viruses. When compared to mock infection, 983 genes were commonly downregulated in both conditions (**Figure 3C1**), and 1259 genes were commonly upregulated (**Figure 3C2**), indicating a largely similar host response with quantitative difference between variants.Furthermore, after ranking the top 10 DEGs by highest FDR in the R77Q vs. WT comparison, we identified a subset of highly regulated genes, the majority of which have established roles in apoptosis, inflammation, DNA damage response, or cytokine signaling (**Supplementary Table 1**).

### Functional enrichment analysis reveals an enriched apoptotic pathway

**Figure 4:**
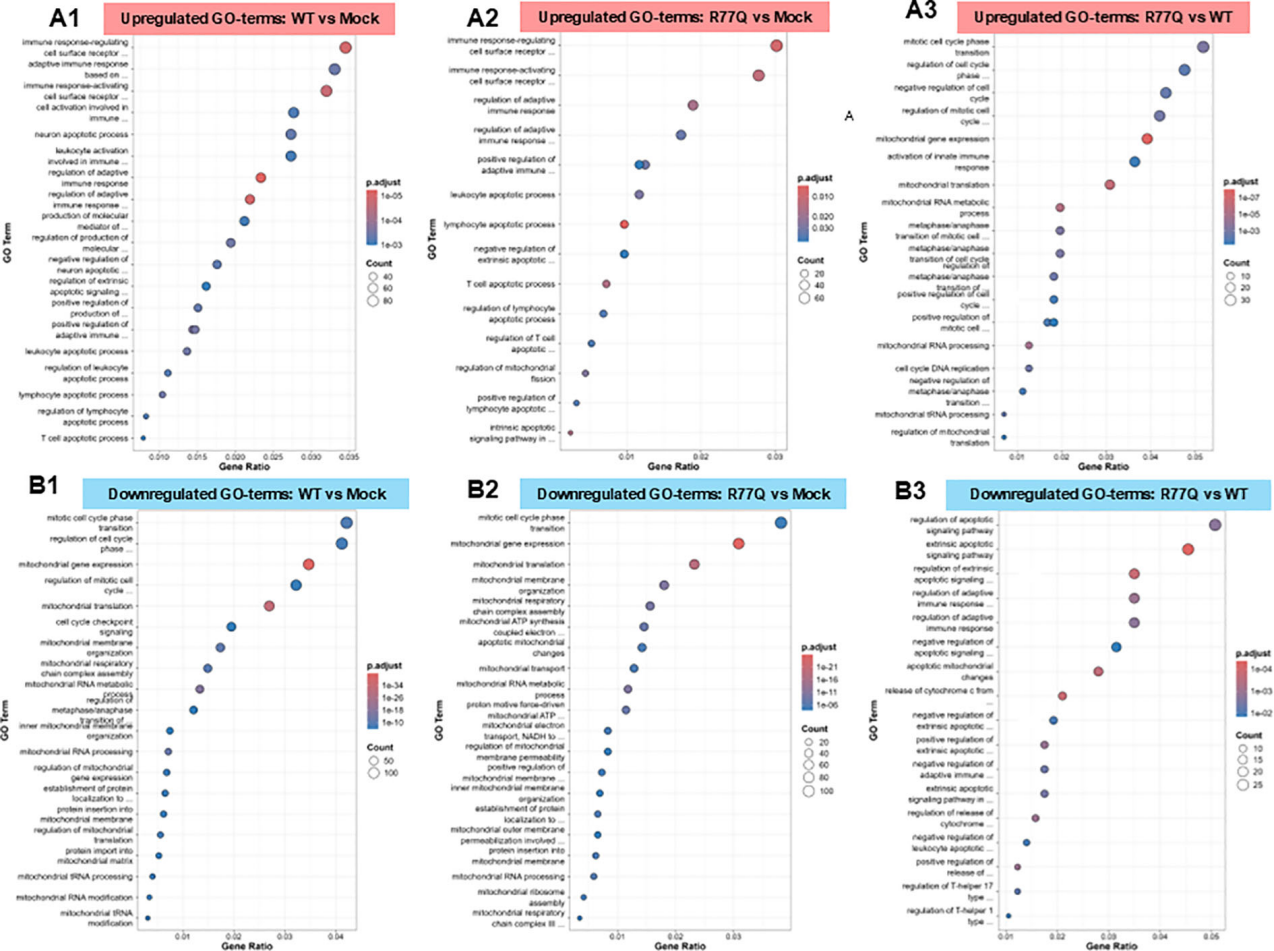
Gene ontology (GO) analysis of Biological Processes (BP) at 72hpi highlight apoptosis-related terms. **(A1–A3)** Dot plot showing the top significantly upregulated apoptosis-related GO terms for WT vs. Mock, R77Q vs. Mock, and R77Q vs. WT respectively. **(B1–B3)** Dot plot showing the top significantly downregulated apoptosis-related GO terms for WT vs. Mock, R77Q vs. Mock, and R77Q vs. WT respectively.

To define biological processes and pathways underlying the transcriptional differences induced by infection with WT virus and the R77Q mutant, we performed GO enrichment and KEGG pathway enrichment analyses using the gprofiler2 R package, version v0.2.4. After filtering GO terms for apoptosis-related terms, analysis of upregulated genes revealed enrichment of apoptosis-associated GO terms in both WT vs. mock and R77Q vs. mock comparisons, suggesting activation of apoptotic transcriptional programs by both Vpr sequences. Notably, WT vs. mock infection was additionally enriched for GO terms related to negative regulation of apoptosis, a feature not observed in the R77Q vs. mock comparison (**Figure 4**) (**Tables 1**, **2**).

**Table 1:** Apoptosis-related GO: BP terms significantly upregulated in the WT vs. mock comparison.

GO: BP term description	Gene ratio	Fold enrichment	zScore	p.adjust
Regulation of adaptive immune response	65/2782	1.967206	6.057433	5.74E-06
Regulation of adaptive immune response based on somatic recombination of immune receptors built from immunoglobulin superfamily domains	61/2782	1.969224	5.875113	1.05E-05
Immune response-regulating cell surface receptor signaling pathway	96/2782	1.690422	5.693436	1.12E-05
Immune response-activating cell surface receptor signaling pathway	89/2782	1.709227	5.595344	1.74E-05
Positive regulation of adaptive immune response based on somatic recombination of immune receptors built from immunoglobulin superfamily domains	40/2782	2.054332	5.056389	2.27E-04
Positive regulation of adaptive immune response	41/2782	2.014139	4.973734	2.80E-04
Leukocyte apoptotic process	38/2782	2.060906	4.950247	3.24E-04
Regulation of production of molecular mediator of immune response	54/2782	1.812287	4.82808	3.28E-04
Lymphocyte apoptotic process	29/2782	2.312936	5.046438	3.28E-04
Adaptive immune response based on somatic recombination of immune receptors built from immunoglobulin superfamily domains	92/2782	1.551481	4.649142	3.82E-04
Neuron apoptotic process	76/2782	1.620209	4.639757	4.46E-04
Positive regulation of production of molecular mediator of immune response	42/2782	1.950208	4.794395	4.51E-04
Negative regulation of neuron apoptotic process	49/2782	1.835279	4.696949	5.19E-04
Regulation of leukocyte apoptotic process	31/2782	2.166579	4.791492	6.10E-04
Regulation of lymphocyte apoptotic process	23/2782	2.474981	4.877916	6.43E-04
Cell activation involved in immune response	77/2782	1.586644	4.465239	7.05E-04
Leukocyte activation involved in immune response	76/2782	1.585312	4.427462	7.96E-04
Production of molecular mediator of immune response	59/2782	1.694824	4.468031	8.31E-04
Regulation of extrinsic apoptotic signaling pathway	45/2782	1.826756	4.463938	0.001008
T cell apoptotic process	22/2782	2.444992	4.702024	0.001036
Negative regulation of immune response	56/2782	1.679825	4.276815	0.001427
Lymphocyte activation involved in immune response	57/2782	1.64434	4.134496	0.002033
Cytokine production involved in immune response	36/2782	1.877343	4.175363	0.002434
Regulation of cytokine production involved in immune response	36/2782	1.877343	4.175363	0.002434
Regulation of neuron apoptotic process	62/2782	1.592107	4.030087	0.002529
Positive regulation of cytokine production involved in immune response	27/2782	2.080011	4.224119	0.002662
Negative regulation of extrinsic apoptotic signaling pathway	30/2782	1.974552	4.125379	0.00309
Regulation of T cell apoptotic process	16/2782	2.522529	4.157635	0.0046
Mature B cell differentiation involved in immune response	13/2782	2.592084	3.864988	0.01018
Extrinsic apoptotic signaling pathway	54/2782	1.531724	3.441164	0.011075
Positive regulation of innate immune response	88/2782	1.380968	3.332059	0.012407
Regulation of immunoglobulin mediated immune response	19/2782	2.077526	3.535002	0.014979
Positive regulation of lymphocyte apoptotic process	9/2782	3.050683	3.816761	0.015688
Intrinsic apoptotic signaling pathway in response to endoplasmic reticulum stress	20/2782	2.02367	3.491575	0.01597
Negative regulation of leukocyte apoptotic process	18/2782	2.103919	3.502444	0.016445
Intrinsic apoptotic signaling pathway in response to hypoxia	6/2782	4.067577	4.036098	0.016445
CD4-positive, alpha-beta T cell differentiation involved in immune response	25/2782	1.8422	3.368387	0.017972
Innate immune response-activating signaling pathway	71/2782	1.407398	3.162688	0.019054
Regulation of apoptotic signaling pathway	82/2782	1.369217	3.128396	0.019853
Alpha-beta T cell activation involved in immune response	25/2782	1.822391	3.307078	0.020192
Alpha-beta T cell differentiation involved in immune response	25/2782	1.822391	3.307078	0.020192
Negative regulation of adaptive immune response	21/2782	1.923854	3.312257	0.021593
Antiviral innate immune response	22/2782	1.887905	3.289596	0.021965
Negative regulation of adaptive immune response based on somatic recombination of immune receptors built from immunoglobulin superfamily domains	20/2782	1.936942	3.266781	0.023982
Negative regulation of lymphocyte apoptotic process	13/2782	2.319233	3.386119	0.024608
Negative regulation of apoptotic signaling pathway	53/2782	1.466541	3.05748	0.025439
T cell differentiation involved in immune response	26/2782	1.745165	3.123419	0.027899
Activation of innate immune response	73/2782	1.367095	2.933546	0.030566
Regulation of extrinsic apoptotic signaling pathway via death domain receptors	15/2782	2.075295	3.135028	0.035785
Immunoglobulin production involved in immunoglobulin-mediated immune response	17/2782	1.953356	3.050792	0.038037
Type 2 immune response	14/2782	2.109114	3.098505	0.038159
Regulation of endoplasmic reticulum stress-induced intrinsic apoptotic signaling pathway	12/2782	2.259765	3.147073	0.038159
Positive regulation of cell cycle G2/M phase transition	11/2782	2.330383	3.133079	0.040452
T cell activation involved in immune response	33/2782	1.57547	2.863249	0.04119
Somatic recombination of immunoglobulin genes involved in immune response	16/2782	1.972159	3.003335	0.041502
Somatic diversification of immunoglobulins involved in immune response	16/2782	1.972159	3.003335	0.041502
Positive regulation of T cell apoptotic process	7/2782	2.965942	3.272397	0.043788
Intrinsic apoptotic signaling pathway	67/2782	1.355859	2.733652	0.044599
T cell mediated immune response to tumor cell	8/2782	2.711718	3.185875	0.045065
Positive regulation of leukocyte apoptotic process	11/2782	2.259765	3.012853	0.049204

**Table 2:** Apoptosis-related GO: BP terms significantly upregulated in the R77Q vs. mock comparison.

Description	Gene ratio	Fold enrichment	zScore	p.adjust
Lymphocyte apoptotic process	24/2484	2.143791	4.116208	0.006813
Immune response-regulating cell surface receptor signaling pathway	75/2484	1.479076	3.698914	0.008935
Immune response-activating cell surface receptor signaling pathway	69/2484	1.484105	3.575935	0.011828
T cell apoptotic process	18/2484	2.240437	3.779232	0.015124
Intrinsic apoptotic signaling pathway in response to hypoxia	6/2484	4.555556	4.38009	0.016012
Regulation of adaptive immune response	47/2484	1.593089	3.477751	0.017012
Regulation of mitochondrial fission	11/2484	2.694146	3.676624	0.026315
Leukocyte apoptotic process	29/2484	1.761481	3.326739	0.028182
Positive regulation of adaptive immune response	31/2484	1.705582	3.23998	0.03156
Regulation of adaptive immune response based on somatic recombination of immune receptors built from immunoglobulin superfamily domains	43/2484	1.554674	3.148031	0.033578
Positive regulation of lymphocyte apoptotic process	8/2484	3.037037	3.549807	0.039571
Regulation of lymphocyte apoptotic process	17/2484	2.048795	3.247489	0.039571
Regulation of T cell apoptotic process	13/2484	2.295435	3.312118	0.04009
Negative regulation of extrinsic apoptotic signaling pathway	24/2484	1.769148	3.048442	0.047009
Positive regulation of adaptive immune response based on somatic recombination of immune receptors built from immunoglobulin superfamily domains	29/2484	1.66807	2.999813	0.04789

Analysis of downregulated GO terms showed shared enrichment for mitochondrial gene expression and cell cycle–related processes in both WT and R77Q infections compared to mock. Direct comparison of R77Q vs. WT revealed upregulation of GO terms associated with intrinsic apoptosis whereas downregulated GO terms were predominantly linked to regulation of extrinsic apoptotic signaling.

**Figure 5:**
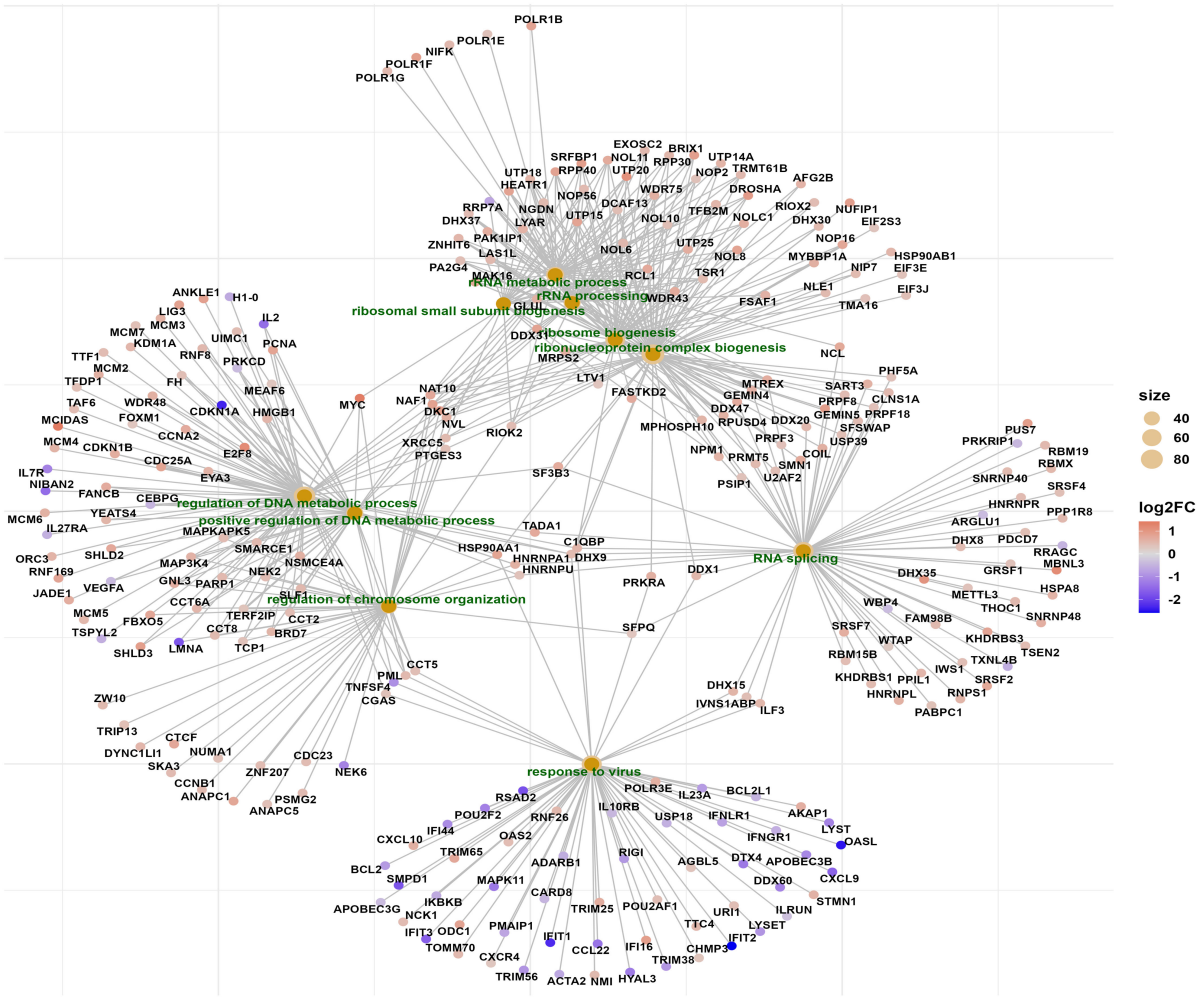
Gene ontology concept network (CNET) of enriched biological processes in the R77Q vs. WT comparison. The CNET plot was generated from significant DEGs (FDR <0.05) in R77Q vs. WT infection at 72 hpi using clusterProfiler. The enriched gene ontology biological processes are denoted by orange nodes, with node size proportional to the number of genes, as indicated by the scale. Additionally, the enriched GO terms are highlighted in green for clarity. Individual gene nodes are colored according to direction of differential expression in R77Q relative to WT, with red indicating upregulated genes and blue indicating downregulated genes. Edges represent gene membership within each functional category.

Our GO gene–concept network analysis of the R77Q vs. WT comparison further resolved functional differences. Among the top 10 enriched themes in the R77Q vs. WT comparison, genes involved in the response to virus were largely downregulated, including multiple antiretroviral restriction factors such as APOBEC and TRIM family members, although select genes involved in viral innate immune response such as IFI16 and RIG1 were upregulated. In contrast, RNA splicing and regulation of DNA metabolic processes were enriched and predominantly upregulated (**Figure 5**).

**Figure 6:**
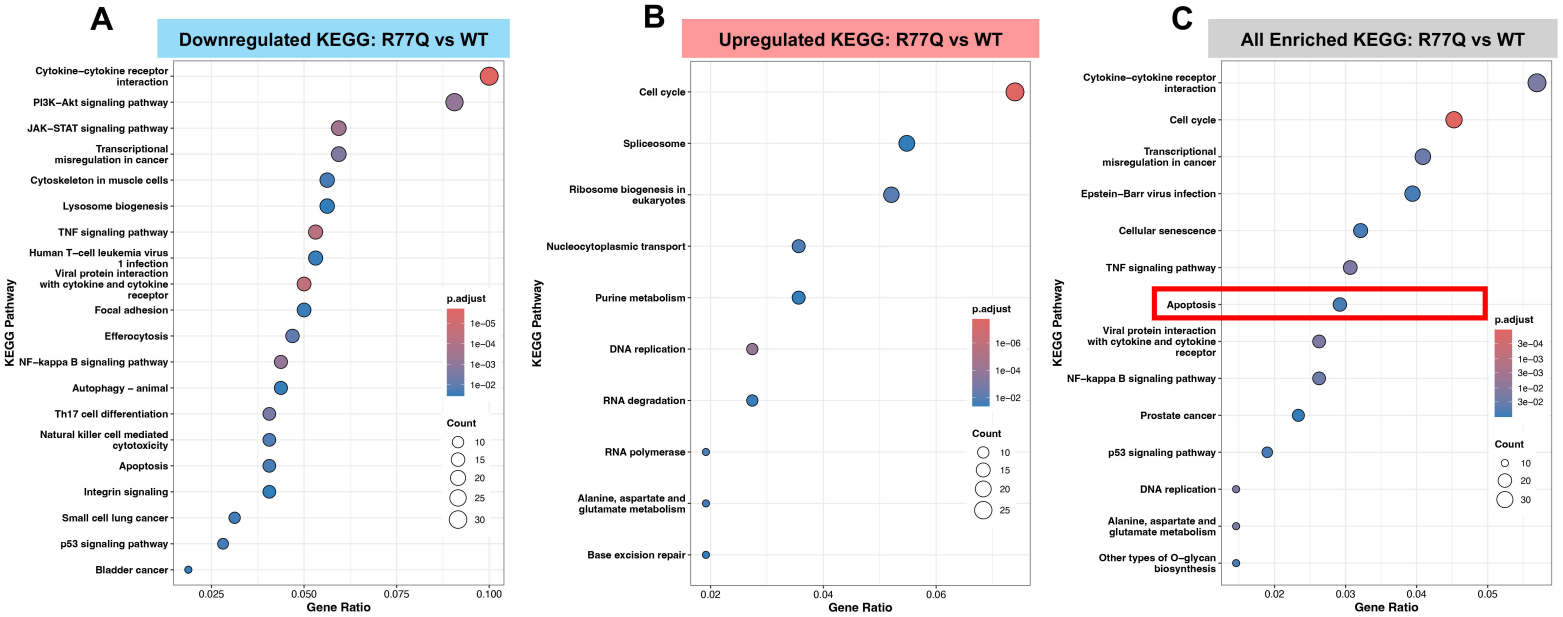
KEGG pathway enrichment analysis of differentially expressed genes in R77Q vs. WT at 72 hpi. KEGG pathway enrichment analysis was performed using gprofiler2 (v0.2.4) on significantly differentially expressed genes (FDR <0.05) identified in the direct comparison between R77Q and WT infection at 72 hpi. Dot plots display enriched KEGG pathways for **(A)** genes downregulated in the R77Q condition relative to WT, **(B)** genes upregulated in R77Q vs. WT, and **(C)** all differentially expressed genes irrespective of direction. Dot size is proportional to the number of genes contributing to each pathway, and dot color reflects the level of statistical significance, as indicated by the scale. The apoptosis pathway is highlighted with a red bounding box and was selected for downstream visualization using Pathview to map differential gene expression onto canonical KEGG apoptosis pathway.

Consistent with these findings, KEGG pathway analysis of the R77Q vs. WT comparison identified downregulation of JAK–STAT, TNF signaling, and apoptotic pathways, while pathways related to cell cycle progression and regulation of DNA metabolic processes were upregulated (**Supplementary Figure 1**). Across all enriched pathways, apoptosis also emerged as a recurrent and significantly enriched process, highlighting apoptotic signaling as an important feature (**Figure 6**).

### R77Q is unable to prevent apoptotic signals at the mitochondrial membrane

**Figure 7:**
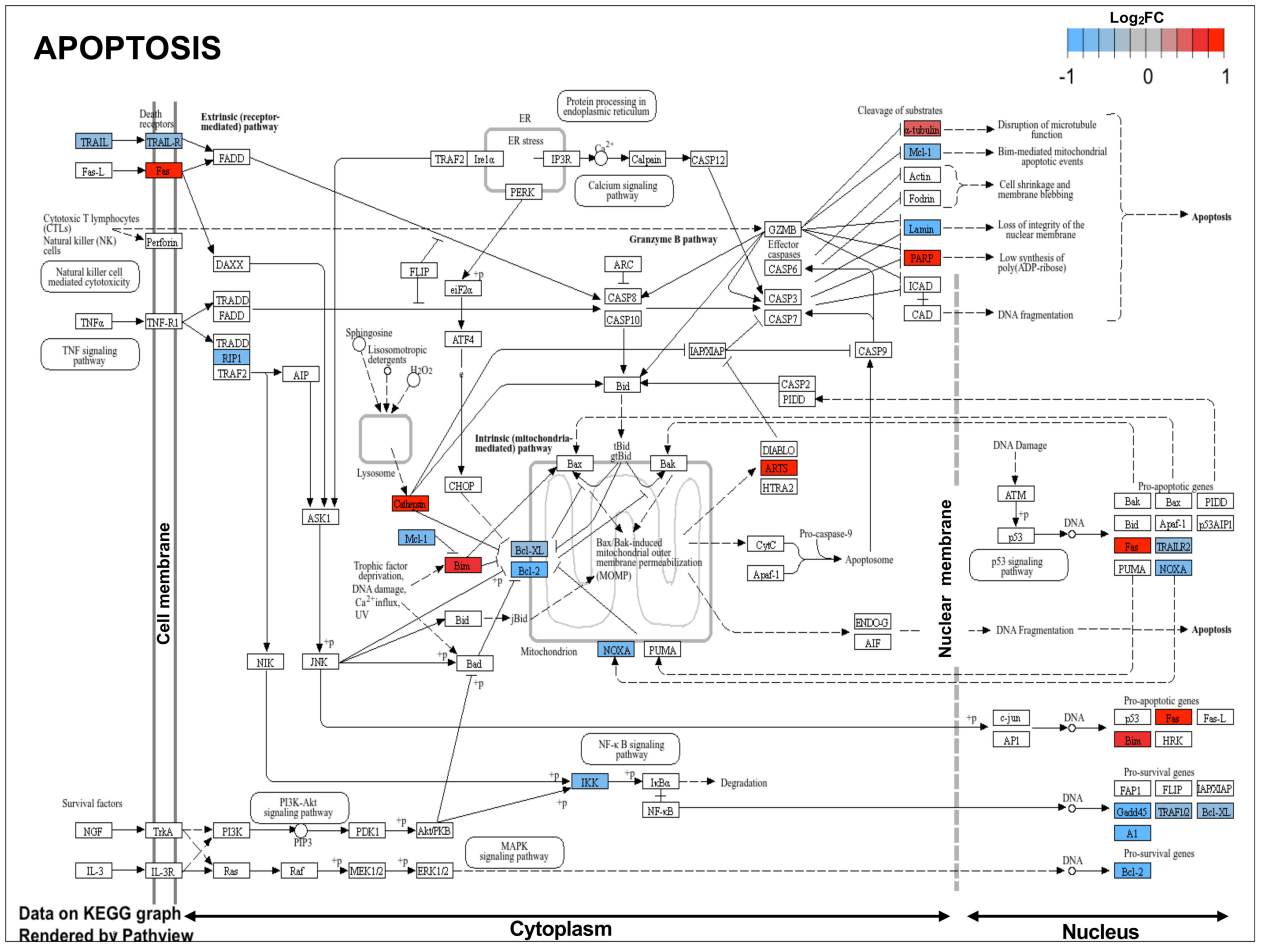
KEGG pathway analysis shows DEGs in the enriched apoptosis pathway in R77Q vs. WT at 72 hpi. The KEGG apoptosis pathway was rendered using Pathview, with differential gene expression data from the R77Q vs. WT comparison at 72 hpi superimposed onto the pathway map. Genes significantly upregulated in the R77Q condition relative to WT are shown in red, whereas genes significantly downregulated are shown in blue. Uncolored pathway components indicate genes that were not differentially expressed in this analysis. This visualization highlights coordinated alterations in apoptotic signaling associated with R77Q infection.

To further define how WT and the R77Q mutant differentially regulate apoptotic signaling, we interrogated the enriched apoptosis pathway using Pathview to overlay differential gene expression onto the KEGG apoptosis map. This analysis revealed coordinated changes in both pro-apoptotic and pro-survival gene expression in the direct R77Q vs. WT comparison (**Figure 7**).

Within the R77Q vs. WT comparison, we observed differentially expressed genes associated with extrinsic apoptotic signaling. *FAS*, which encodes the death receptor Fas that initiates extrinsic apoptosis upon ligand engagement, was significantly upregulated in R77Q-infected cells relative to WT (logFC = 1.13; FDR = 9.78 × 10^−5^). In contrast, *TNFSF10* (*TRAIL*), encoding a pro-apoptotic death ligand, and the receptor *TNFRSF10* were downregulated (logFC = −0.57; FDR = 0.00226 and logFC = −0.551; FDR = 0.00688, respectively). Despite these changes at the receptor–ligand level, downstream components required to propagate the extrinsic apoptotic cascade were not enriched, consistent with GO analyses indicating overall suppression of extrinsic apoptotic signaling in the R77Q vs. WT comparison (**Figure 4**).

In contrast, pronounced differences were observed in the regulation of intrinsic apoptosis–associated genes between WT and R77Q infection. Several anti-apoptotic genes of the *BCL2* family were downregulated, while pro-apoptotic regulators were enriched. Notably, the anti-apoptotic gene *BCL2*, which encodes the BCL-2 protein that inhibits ANT1-mediated mitochondrial pore formation and cytochrome c release (Zhang et al., 2002; Andersen et al., 2006; Wallet et al., 2020), was significantly upregulated in WT-infected cells (logFC = 1.03; FDR = 7.39 × 10^−4^) but not in R77Q-infected cells (logFC = 0.20; FDR = 0.517). Direct comparison confirmed significantly reduced *BCL2* expression in R77Q relative to WT infection (logFC = −0.83; FDR = 0.0225). Conversely, *BCL2L11* (*bim*), a pro-apoptotic gene that encodes a BH3-only protein (Bim) that is activated in response to DNA damage and functions as a potent antagonist of BCL-2–mediated survival signaling, was significantly upregulated in R77Q-infected cells relative to WT (logFC = 0.709; FDR = 0.0256).

Taken together, these data indicate a coordinated downregulation of anti-apoptotic gene expression at the mitochondrial membrane in R77Q-infected cells. In contrast to WT, which retains the ability to induce *BCL2* expression and reinforce mitochondrial survival signaling, the R77Q mutant fails to mount this anti-apoptotic response, suggesting the potential for a loss-of-function phenotype in the regulation of mitochondrial apoptotic pathways by the R77Q mutant relative to the WT virus.

### Mitochondrial membrane potential is reduced in R77Q-infected cells

**Figure 8:**
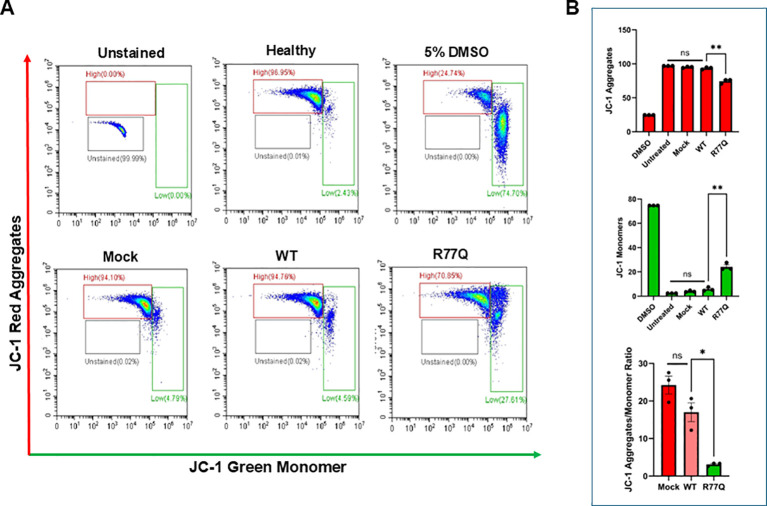
Mitochondrial membrane potential (ΔΨm) is reduced in R77Q-infected cells. **(A)** Representative flow cytometry plots of JC-1 fluorescence in HUT78 cells following infection with WT or HIV-1 Vpr R77Q mutant, 72 hours post infection. JC-1 aggregates (red fluorescence) indicate polarized mitochondrial membrane (high ΔΨm), whereas monomeric JC-1 (green fluorescence) indicates depolarized mitochondrial membrane (low ΔΨm). Cells treated with 5% DMSO for 72 hours were used as positive control for mitochondrial depolarization. Staining and acquisition of the control and samples were performed under identical conditions. **(B)** Quantification of mitochondrial membrane potential shown as JC-1 aggregates (red), JC-1 monomer (green) and the JC-1 aggregate to monomer ratio. Data represents mean and the error bars are standard error of mean from 3 biological replicates. * represents *p* ≤ 0.05, ** represents *p* ≤ 0.01.

To evaluate mitochondrial membrane integrity following infection, mitochondrial membrane potential (ΔΨm), an early hallmark of intrinsic apoptosis, was assessed using the JC-1 assay in HUT78 cells infected with either WT or R77Q virus as described previously. JC-1 dye is a membrane-potential sensitive dye that exhibits a fluorescence emission shift depending on mitochondrial polarization. In depolarized mitochondria, JC-1 remains in its monomeric form, emitting green fluorescence with a peak at ~529-530nm. In contrast, in polarized mitochondria, JC-1 accumulates and forms J-aggregates that emit red fluorescence with a peak at ~590 nm. At 72 hpi, R77Q-infected cells exhibited a pronounced shift from red to green fluorescence relative to WT-infected cells, indicating a loss of ΔΨm (**Figure 8A**). Quantification of the red/green fluorescence ratio confirmed a significant reduction in ΔΨm in R77Q-infected cells compared to WT-infected cells (p ≤ 0.05) (**Figure 8B**). This depolarization is consistent with mitochondrial dysfunction and supports enhanced activation of intrinsic apoptotic pathways in the presence of the R77Q mutation.

### Protein–protein interaction analysis identifies BCL-2 family–associated networks

**Figure 9:**
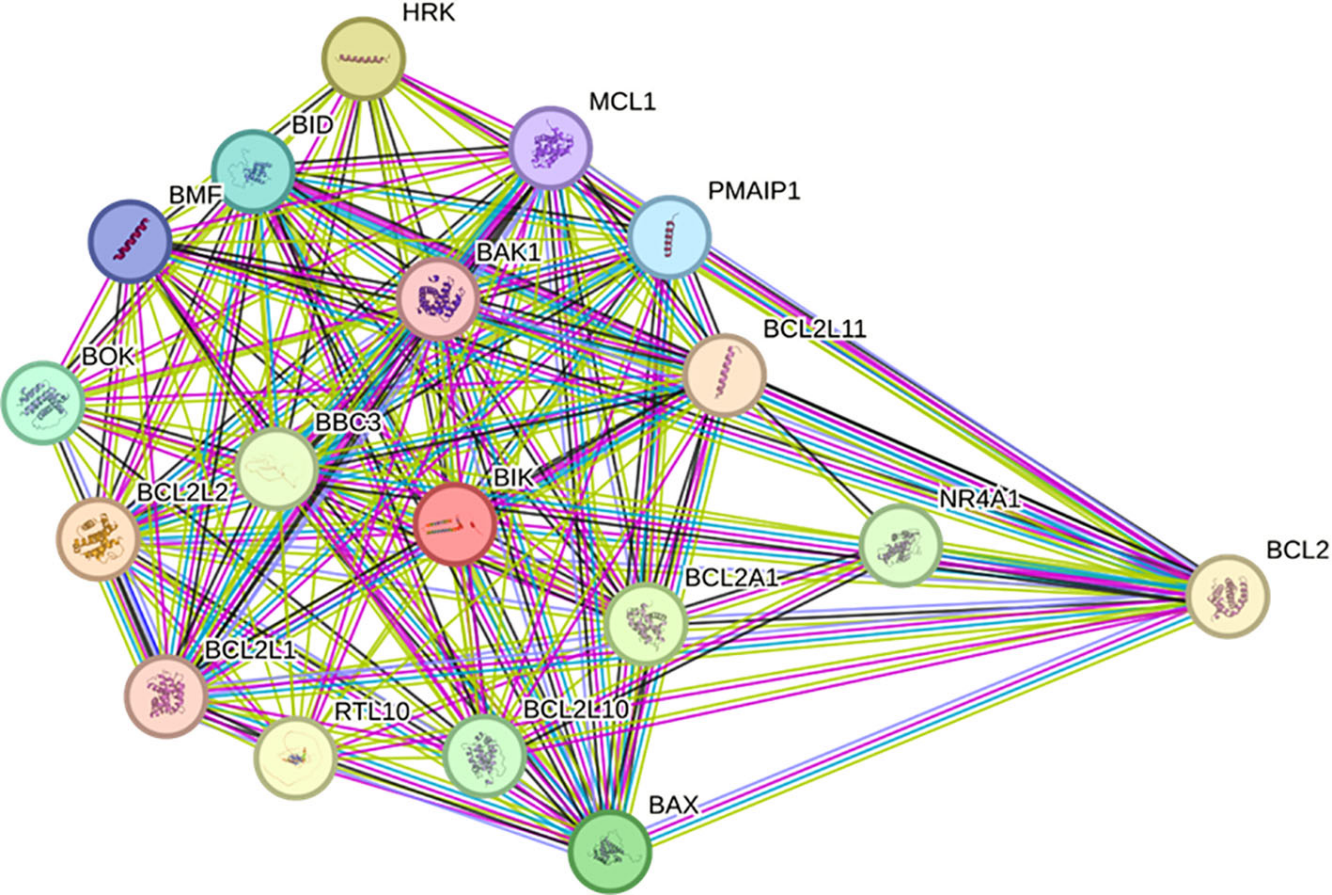
STRING protein-protein interaction analysis of the BCL-2 family local network cluster in R77Q vs. WT at 72hp. The protein-protein interaction (PPI) analysis was performed using STRING to examine interactions among differentially regulated BCL-2 family members. Each node represents an individual protein, and edges represent known or predicted physical or functional interactions between proteins. The local network cluster is comprised of 18 interacting proteins (nodes), compared with proteins expected by chance, indicating substantial enrichment. The average node degree of 15.2 reflects the high number of interactions per protein within the cluster, while the average local clustering coefficient of 0.96 indicates that proteins are not only highly connected to a central network but also strongly interconnected with one another. Consistent with this, the PPI enrichment test was significant (p < 1 × 10^-16^), supporting a nonrandom, functionally coherent interaction network among differentially regulated BCL-2 family members.

To further assess whether the transcriptional differences observed between the R77Q mutant and the WT virus converge on coordinated protein networks, we performed protein–protein interaction (PPI) analysis using the Search Tool for the Retrieval of Interacting Genes/Proteins (STRING) database on differentially expressed genes (FDR < 0.05) from the R77Q vs. WT comparison at 72 hpi. This analysis revealed a highly interconnected interaction network, consisting of 18 nodes and 137 edges. Nodes represent individual proteins and edges represent known or predicted functional interactions. The resulting 137 edges substantially exceeds the expected number of interactions for a random gene set of comparable size (expected edges = 3). The resulting network exhibited a high average node degree (15.2), which represents the average number of interaction partners associated with a given protein in the network with a strong tendency toward clustering (average local clustering coefficient = 0.96), indicating extensive functional connectivity among these proteins. Consistent with this structure, the STRING PPI enrichment analysis was highly significant (p < 1 × 10^−16^), supporting the conclusion that these proteins form a biologically coherent interaction network rather than a random assembly (**Figure 9**).

Notably, the interaction network was dominated by proteins associated with mitochondrial apoptosis and BCL-2 family–mediated regulation of cell death, consistent with the transcriptional enrichment of apoptotic pathways observed in GO and KEGG analyses. The resulting network was centered on regulators of mitochondrial apoptosis, with hub nodes including BCL-2 family members and associated apoptotic effectors. Prominent hubs included the anti-apoptotic proteins BCL-2, Bcl-xL (gene name *BCL2L1*), Bcl-w (*BCL2L2*), and Mcl-1, alongside pro-apoptotic factors Bax, Bim (*BCL2L11*), Bid, Bmf, Bok, Hrk, Puma (*BBC3*), and Noxa (*PMAIP1*). In addition, NR4A1, a nuclear receptor known to modulate BCL-2–dependent apoptotic signaling through mitochondrial translocation (Liu et al., 2019), was identified as a significantly connected node. The prominence of both pro- and anti-apoptotic regulators within this network highlights extensive crosstalk at the mitochondrial membrane. Several highly connected nodes corresponded to key regulators of mitochondrial membrane integrity and apoptotic signaling, reinforcing the notion that differential regulation of apoptosis-related genes in the presence of the R77Q mutant reflects coordinated disruption of protein interaction networks rather than isolated transcriptional changes.

### BCL-2 is upregulated at a protein level in WT-infected cells

**Figure 10:**
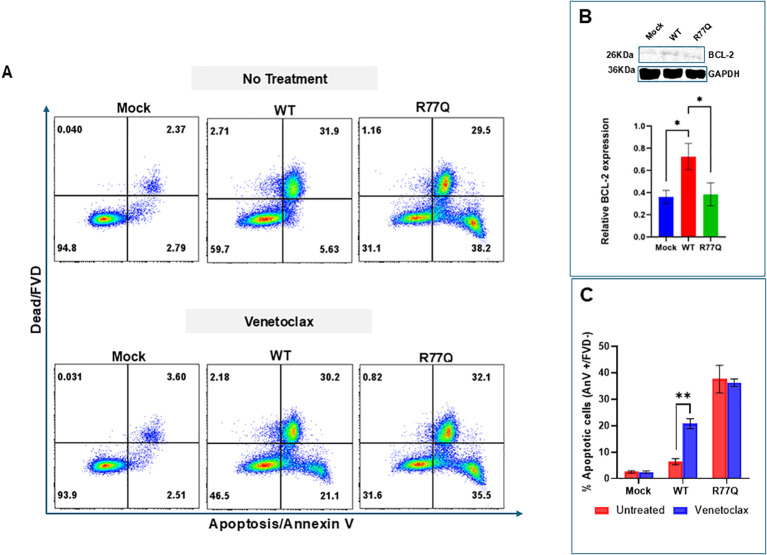
BCL-2 inhibition by Venetoclax shifts WT-induced cell death toward apoptosis. **(A)** Representative flow cytometry plots of Annexin V and Fixable Viability dye staining of HUT78 cells following infection with WT or HIV Vpr R77Q mutant in the presence or absence of 200nM Venetoclax. **(B)** Immunoblot analysis of BCL-2 expression in WT and R77Q infected cells, 72 hours post infection at 0.3 MOI. Bar graph shows relative BCL-2 expression was normalized with GAPDH. **(C)** Bar graph shows percentage of apoptosis in WT-infected and R77Q-infected cells in the presence or absence of Venetoclax. Data represents 3 biological replicates.

To validate the transcriptional upregulation of *BCL2* at a protein level, immunoblot analysis was performed using lysates from cells infected under the same conditions as those used for RNA-seq. Densitometry quantification of BCL-2 expression was normalized to GAPDH. WT-infected cells exhibited increased BCL-2 protein expression, with a 1.8-fold increase relative to R77Q-infected cells (p=0.0322) and a 2-fold increase compared to mock controls (p=0.0139). Although BCL-2 expression was modestly increased in R77Q-infected cells relative to mock controls, this difference did not reach statistical significance (p=0.725) (**Figure 10B**). These data support the transcriptomic data and suggest that WT infection upregulates BCL-2, whereas R77Q fails to mount a comparable response.

### Modulation of BCL-2 alters apoptosis in R77Q- and WT-infected cells

**Figure 11:**
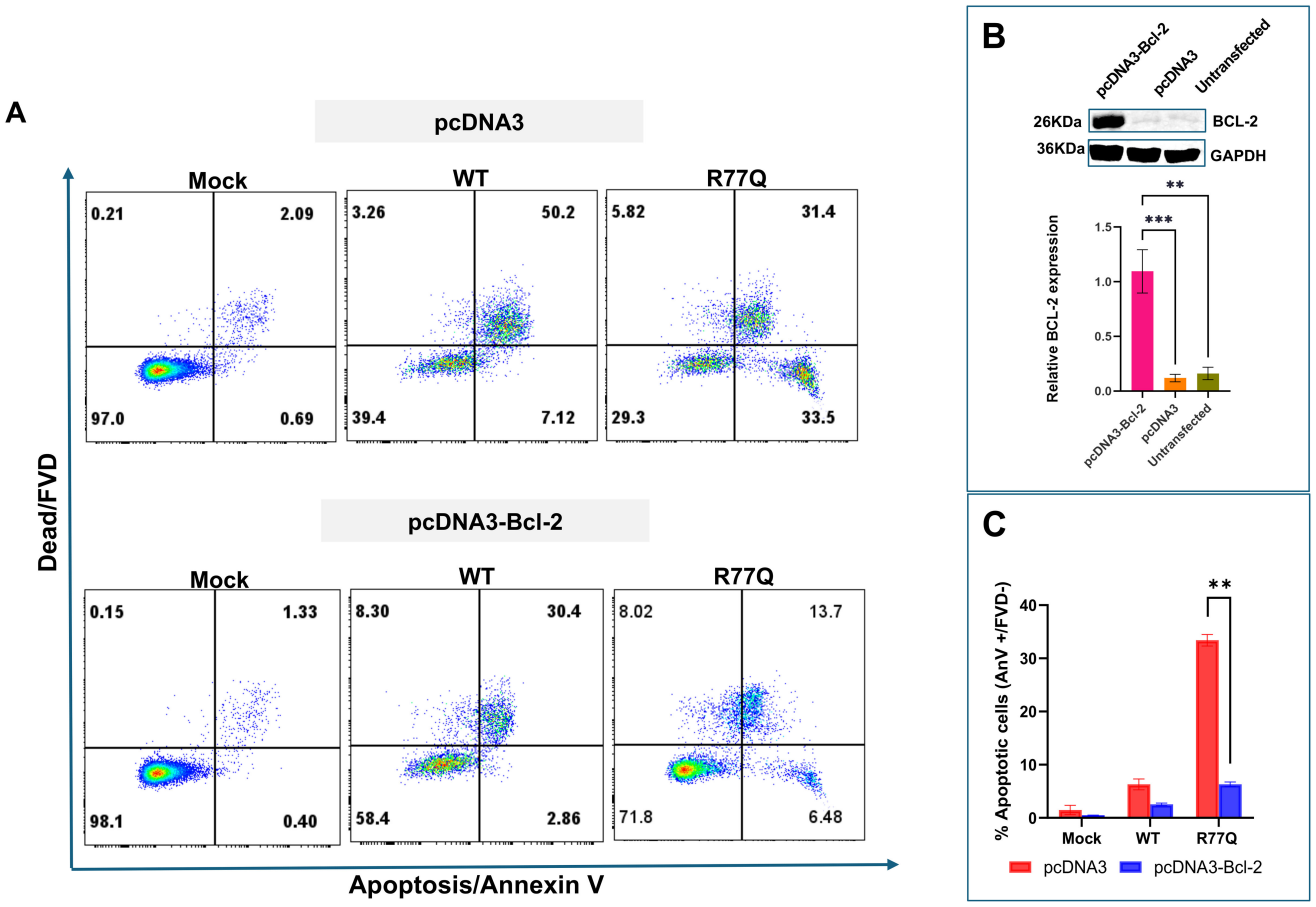
Bcl-2 overexpression attenuates apoptosis in R77Q-infected cells. **(A)** Representative flow cytometry plots of Annexin V and fixable viability dye staining in HUT78 cells following infection with either WT or HIV Vpr R77Q mutant in cells transfected with pcDNA3-Bcl-2 (*BCL2* overexpression) or pcDNA3 (empty vector). **(B)** immunoblot analysis of BCL-2 expression following stable transfection with pcDNA3-Bcl-2 or the empty vector. Relative quantification of BCL-2 protein levels, normalized to GAPDH, was performed using ImageJ software.

To determine whether BCL-2 contributes to the regulation of apoptosis in this system, we ectopically overexpressed in HUT78 cells prior to infection and assessed the resulting effects on cell death. Successful transfection was confirmed by immunoblot analysis, which demonstrated an approximately 10-fold increase in BCL-2 protein levels relative to empty vector or untransfected controls (**Figure 11B**). Following infection, overexpression of BCL-2 markedly reduced apoptosis in R77Q-infected cells at 72 hpi, consistent with a role for BCL-2 in suppressing R77Q-induced apoptosis. We next examined whether pharmacological inhibition of BCL-2 would have the opposite effect. Cells were treated with Venetoclax, a selective BCL-2 inhibitor, prior to infection with WT or R77Q mutant. Notably, inhibition of BCL-2 in WT-infected cells resulted in a shift in the predominant mode of cell death from necrosis to apoptosis (**Figures 10A, C**), suggesting that BCL-2 activity influences the balance between distinct cell death pathways during HIV-1 infection.

## Discussion

HIV-1 Vpr polymorphisms are linked to differential rates of HIV-1 disease progression, highlighting how subtle viral sequence variations can translate into meaningful biological outcomes (Emiliani et al., 1996; Chandhasin et al., 2005; Goodman et al., 2007; Gianella et al., 2011). Among these, the Vpr R77Q mutation has been consistently associated with delayed progression to AIDS, yet the mechanistic basis for this phenotype has remained unclear (Jacquot et al., 2009). Earlier studies proposed reduced cell death in R77Q infection as the sole basis for the LTNP phenotype (Fischer et al., 2004; Mologni et al., 2006; Jacquot et al., 2009; Saxena et al., 2018). However, subsequent work by our group demonstrated that this difference is not just solely quantitative but also qualitative, with R77Q preferentially inducing apoptotic rather than necrotic forms of cell death (Solis-Leal et al., 2024). This distinction is biologically significant (Shen et al., 2023; Vince et al., 2025), as apoptosis is comparatively non-inflammatory (Terai et al., 1991; Hetts, 1998; van Delft et al., 2010), whereas necrotic cell death promotes chronic inflammation (Mohammadi et al., 2013; Gao et al., 2024; Thirion et al., 2025; Vince et al., 2025), a hallmark of HIV-1 disease progression (Lawn et al., 2001; Deeks et al., 2013). Despite these observations, the molecular mechanisms linking R77Q-mediated modulation of cell death to alter disease progression remains largely unresolved (Thirion et al., 2025). In this study, we sought to delineate the host transcriptional mechanism underlying R77Q-mediated modulation of apoptotic signaling.

Using transcriptomic profiling, we identified several key features of the host response to R77Q vs. WT infection. Both WT and R77Q triggered clear transcriptional changes relative to mock-infected cells as early as 4 hpi. By 72 hpi, strain specific differences emerged, with nearly 300 DEGs distinguishing R77Q from WT infection at this time point. Functional enrichment analysis identified that apoptosis and apoptosis-related GO-terms were enriched in both R77Q and WT infections; however, WT infection was uniquely associated with downregulation of intrinsic apoptotic GO-terms, a pattern not shown in R77Q infection. Furthermore, KEGG analysis revealed significant enrichment of apoptotic signaling in the R77Q vs. WT comparison and, as visualized by Pathview (Luo and Brouwer, 2013), highlighted a failure of R77Q infection to upregulate key anti-apoptotic regulators such as *BCL2*, suggesting a potential loss of function phenotype. Protein-protein interaction analysis revealed a highly significant interaction network centered on the BCL-2 family of proteins, suggesting coordinated regulation of apoptotic signaling involving this network. Importantly, these transcriptional differences were supported at both the protein and functional levels. BCL-2 expression was significantly elevated in WT-infected cells compared to R77Q-infected cells. Consistent with impaired anti-apoptotic signaling, R77Q-infected cells exhibited reduced ΔΨm, as demonstrated by the JC-1 fluorescence shift, indicative of mitochondrial depolarization and suggesting activation of intrinsic apoptotic pathways. Furthermore, direct modulation of BCL-2 confirmed its central role in determining cell fate: overexpression attenuated apoptosis in R77Q-infected cells, whereas, pharmacological inhibition shifted WT-induced cell death from necrosis toward apoptosis.

During the early phase of infection (4, 8, 12, 24 hpi), both R77Q and WT-infected cells showed clear transcriptomic changes relative to mock-infected cells, consistent with previous work by others (Mohammadi et al., 2013; Bauby et al., 2021). Bauby et al. (2021) demonstrated that HIV-1 infection triggers rapid and widespread transcriptomic changes in CD4+ T cells as early as 4–12 hpi, with Vpr driving much of this early host response (Bauby et al., 2021). Similarly, Mohammadi et al, reported broad and rapid host gene expression changes following HIV-1 infection, underscoring the magnitude of early virus-driven transcriptional disruptions (Mohammadi et al., 2013). Notably, early time points are characterized by extensive differential expression when compared to uninfected controls (Coelho et al., 2021). In contrast, direct comparison between closely related viral strains may reveal far less pronounced differences during the early infection (Wu et al., 2024). Taken together, these observations suggest that the early transcriptional landscape is dominated by a broad, generalized host response to infection, whereas strain-specific effects become more discernible at later stages (Moni et al., 2013).

In our model, no significant DEGs were detected when directly comparing R77Q vs. WT at early time points. This was expected given the nearly identical viral genomes (~99.99% identity) and the use of a relatively low multiplicity of infection (MOI = 0.3) in this model, conditions under which strain-specific effects often emerge more slowly (Russell et al., 2018; Wang et al., 2024). Despite the subtlety of the R77Q mutation, the magnitude of transcriptional changes observed at 72 hpi was striking. This finding aligns with prior work demonstrating that the *vpr* gene can exert significant effects on host gene expression (Díez-Fuertes et al., 2019; Sandoval et al., 2024). Consistent with this, Imbeault et al. (2012) showed that small viral mutations can yield only minor transcriptomic differences compared to WT infection. In their experiment model, at 72 hpi, HIV-1 infection generated more than 3,300 DEGs relative to mock, yet one of the *vpu* point mutants differed from WT by only about 7 DEGs at the same time point, a pattern expected given the similarity of the two viral strains (Langer et al., 2019). It should be noted that transcriptional changes detected at this later stage may, in part, reflect secondary host responses arising from cumulative viral perturbation rather than immediate early effects of the mutation. Nonetheless, this time point represents the earliest stage at which consistent and reproducible strain-specific differences were detected in our model.

Building on these global transcriptional differences, a closer inspection of the DEGs revealed a consistent enrichment of apoptosis-related genes, in line with the established role of Vpr as a regulator of programmed cell death (Andersen and Planelles, 2005; Ma et al., 2012). Within the extrinsic apoptotic pathway, R77Q-infected cells showed selective modulation of death receptor signaling. *FAS*, which encodes the Fas death receptor responsible for initiating the extrinsic apoptosis pathway upon FasL engagement (Mueller et al., 2001; Arokium et al., 2009; Hu et al., 2025), was significantly upregulated in R77Q vs. WT-infected cells. However, *FasL* was downregulated, suggesting that, at least at the gene expression level, although receptor expression increased, the corresponding ligand signal required to trigger the pathway was reduced. This imbalance suggests that the extrinsic pathway was primed but not activated (López-Huertas et al., 2013).

In the intrinsic apoptotic pathway, across both infections, the DEG findings support a strong intrinsic apoptotic pressure, but we noted a key difference in how effectively each viral strain engages the anti-apoptotic *BCL2* family of genes. Prior work has shown that Vpr can directly and indirectly interact with the mitochondria and promotes intrinsic, caspase-independent apoptosis through mitochondrial permeabilization and *BCL2* downregulation (Roumier et al., 2002; Andersen et al., 2006). Consistent with that framework, our DEGs at 72 hpi point to a stressed, intrinsic apoptotic condition in both R77Q and WT infections, yet with different survival responses.

In WT-infected cells, anti-apoptotic gene expression is preserved. Compared to R77Q, WT infection shows higher expression of multiple anti-apoptotic factors in the *BCL2* family including *BCL2*, *bcl2l1/bcl-xl* and *mcl1*. This pattern fits the broader HIV-1 literature in which infected cells can simultaneously experience pro-death signals while maintaining survival programs that delay mitochondrial outer membrane permeabilization (MOP), supporting continued viral production or necrotic cell death (Krajewski et al., 1997; Quaye et al., 2025). R77Q, by contrast, shows a failure to induce this anti-apoptotic response. The most striking counterweight is the selective significant increase in the BH3-only activator *BCL2L11* (Bim), a potent antagonist of *BCL2* that lowers the threshold for MOP (O’Connor, 1998). At a functional level, this divergence was further supported by mitochondrial membrane integrity assay, where R77Q infection exhibited reduced ΔΨm, as measured by JC-1 fluorescence. JC-1 has been widely used to assess mitochondrial membrane depolarization as a hallmark of intrinsic apoptosis (Sternfeld et al., 2009; De Simone et al., 2016; Blagov et al., 2023), and the observed shift in fluorescence is consistent with mitochondrial depolarization. While our data support a predominant role for intrinsic apoptosis in the R77Q phenotype, mixed regulations of extrinsic pathway components suggest additional contributions from pathway crosstalk or partial activation, highlighting the complexity of HIV-1-induced cell death regulation (Aillet et al., 1998).

In clinical settings, HIV-1 infection has been associated with increased expression of the anti-apoptotic protein, BCL-2 (Airò et al., 2000; Ren et al., 2020; Chandrasekar and Badley, 2022). Additionally, in multiple different contexts, increased BCL-2 expression in HIV-1 positive vs. HIV-1 negative CD4+ T cells have been reported (Strack et al., 1996; Zhang et al., 2002; Chandrasekar et al., 2019). Consistent with these observations, our protein-level analysis demonstrated that WT-infected cells exhibit elevated BCL-2 expression, as determined by immunoblotting, supporting the notion that HIV-1 can maintain survival signaling despite concurrent pro-apoptotic stress. While post-transcriptional and post-translational regulation of *BCL2* is well documented and likely to contribute to these effects (Letai and Kutuk, 2008; Willimott and Wagner, 2010), the concordance between our transcriptomic and protein-level data strengthens the interpretation that WT infection actively engages anti-apoptotic pathways. In contrast, R77Q-infected cells failed to upregulate BCL-2 beyond mock levels, a finding that aligns with the observed apoptotic phenotype (Solis-Leal et al., 2024).

Our transcriptomic analyses revealed that *BCL2* was significantly upregulated in WT-infected cells, whereas R77Q infection failed to elevate *BCL2* expression beyond baseline, suggesting differential engagement of anti-apoptotic signaling at the gene level. This divergence was reflected at the protein-level, where BCL-2 expression was significantly higher in WT-infected cells compared to R77Q-infected cells. To directly interrogate the role of BCL-2 in regulating apoptosis in this system, we modulated its expression and assessed the resulting effect on cell death. Ectopic overexpression of BCL-2, confirmed by an approximately 10-fold increase in protein levels, significantly attenuated apoptosis in R77Q-infected cells, suggesting a direct role for BCL-2 in suppressing the apoptotic phenotype associated with this variant. Conversely, pharmacological inhibition of BCL-2 using Venetoclax shifted WT-induced cell death from necrosis towards apoptosis, suggesting that BCL-2 not only promotes cell survival but also influences the mode of cell death during HIV-1 infection.

Taken together, these findings support a model in which both WT and R77Q infections impose apoptotic stress but differ in their capacity to mount a compensatory survival response. WT infection preserves mitochondrial integrity through BCL-2 upregulation, whereas R77Q lacks this response, shifting the balance toward mitochondrial membrane permeabilization and execution of apoptosis. This adds a novel mechanistic layer to prior observations that the apoptotic phenotype of Vpr is highly context- and sequence-dependent. In this context, the R77Q phenotype is suggestive of a loss of pro-survival signaling rather than a gain of proapoptotic function, consistent with the BCL-2 immunoblot analysis.

This results align with growing interest in leveraging cell-death pathways as therapeutic strategies in HIV-1 infection (Badley et al., 2013). Inhibition of BCL-2 has been proposed as a means to selectively sensitize infected cells to apoptosis and prevent viral persistence (Reece et al., 2022; Zhou et al., 2023). Venetoclax, an FDA-approved, selective BCL-2 inhibitor used in hematological malignancies, disrupt BCL-2 -mediated survival signaling by antagonizing its interaction with proapoptotic proteins (Mihalyova et al., 2018; Lasica and Anderson, 2021). In preclinical HIV-1 models, Venetoclax has been shown to reduce the viral reservoir and is currently under clinical evaluation (University of Aarhus, 2025). Our findings provide a mechanistic support for this approach, demonstrating that modulation of BCL-2 can influence both the extent and mode of cell death, including a shift from necrotic to apoptotic pathways that may mitigate inflammatory consequences of infection.

We recognize that there are some shortcomings related to our experimental approach, such as the use of a CD4+ T cell line instead of the use of primary CD4+ T cells which would be more physiologically relevant. While we previously showed that primary CD4+ T cells are similarly induced into an apoptotic pathway following infection with R77Q virus (Solis-Leal et al., 2024), our approach did not allow us to examine the transcriptional response in these cells. Another shortcoming is that many genes exhibit gene expression regulation patterns that are not expected to be fully detectable at the level of mRNA expression, the focus of this study. While we have validated expression of BCL-2 at the protein level, it is beyond the scope of this study to validate all DEGs in similar fashion. As with any transcriptomic study, our data do not fully capture post-transcriptional regulation (Díez-Fuertes et al., 2019), therefore, we envision that in the future, protein-level and mechanistic experiments will be important to fully capture the host response to the R77Q mutant. Importantly, the strengths of this approach lie in providing an unbiased, genome-wide view of how a single Vpr polymorphism reshapes host-cell fate, which is an essential foundation before dissecting downstream functional steps. Although *in vitro* systems cannot reproduce every aspect of *in vivo* infection, they offer the controlled environment necessary to reveal the distinct apoptotic mechanism induced by the R77Q mutant. Taken together, these design choices provide a rigorous and interpretable framework for understanding how the R77Q mutant influences apoptotic signaling and HIV-1 pathogenesis, setting the stage for more targeted mechanistic studies.

## Conclusion

In summary, these data support a model in which both viruses impose intrinsic apoptotic stress, but only WT infection elicits an anti-apoptotic response that prevents apoptosis. R77Q lacks this mechanism, making infected cells more susceptible to apoptosis once stress accumulates. The novel element of our findings is the demonstration that R77Q’s apoptotic phenotype arises not from a uniquely stronger pro-death signal, but potentially from a loss-of-function phenotype that could prevent the upregulation of key anti-apoptotic genes. This mechanistic distinction provides a potential explanation for the reduced inflammatory profile linked to R77Q infection and offers insight into its association with slower disease progression.

## Data Availability

The datasets presented in this study can be found in online repositories. The names of the repository/repositories and accession number(s) can be found in the article/**Supplementary Material**.

## Supplementary material

Supplementary Figure 1 KEGG Cell Cycle pathway enrichment in R77Q vs. WT at 72 hpi. The KEGG cell cycle pathway was visualized using Pathview, with differential gene expression data from the R77Q vs. WT comparison at 72 hpi overlaid onto the pathway map. Genes significantly upregulated in R77Q relative to WT are shown in red, while significantly downregulated genes are shown in blue. Uncolored nodes indicate genes that were not differentially expressed. The predominance of red nodes reflects broad upregulation of cell cycle–associated genes in R77Q compared to WT infection.

Supplementary Figure 2 TNF signaling pathway analysis in R77Q vs. WT at 72 hpi. The KEGG TNF signaling pathway was visualized using Pathview, with differential gene expression data from the R77Q vs. WT comparison at 72 hpi overlaid onto the pathway map. Genes significantly upregulated in R77Q relative to WT are shown in red, whereas significantly downregulated genes are shown in blue. Uncolored nodes represent genes that were not differentially expressed. The predominance of blue nodes indicates broad downregulation of TNF signaling components in R77Q compared to WT infection.

Supplementary Table 1 Top 10 most significant DEGs filtered in the R77Q vs. WT comparison.
