# Supplementary figures and images for: The HIV-1 Vpr R77Q mutant alters host apoptotic gene regulation in CD4+ T cells
Source: Front Cell Infect Microbiol. 2026 Jun 29;16:1830094. doi: 10.3389/fcimb.2026.1830094 (PMC13357995; doi:10.3389/fcimb.2026.1830094)

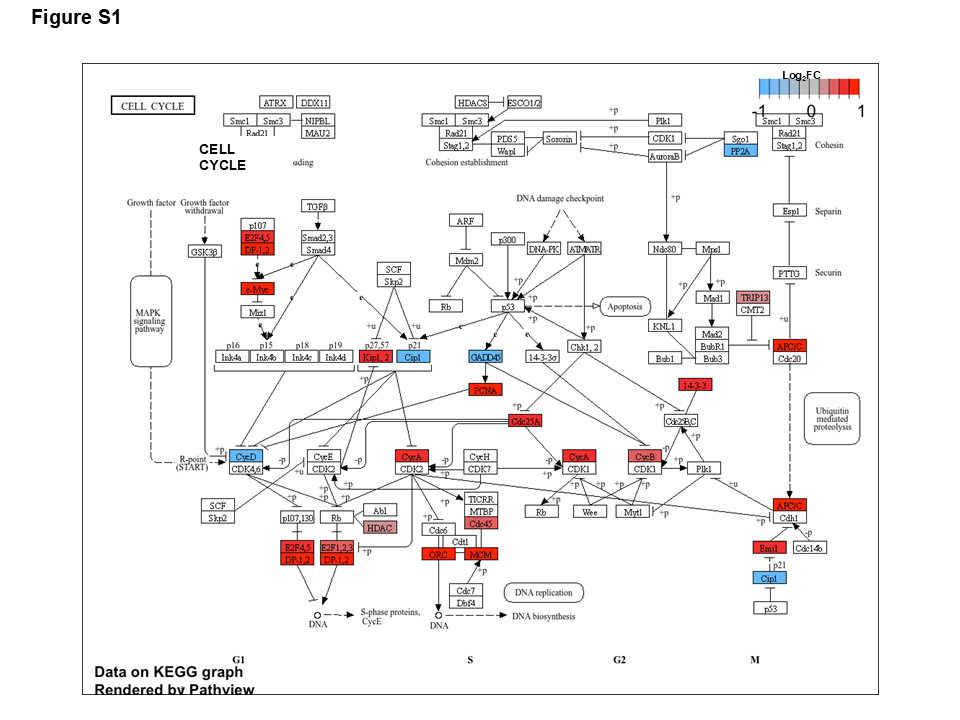

Supplement: Supplementary Figure 1 — KEGG Cell Cycle pathway enrichment in R77Q vs. WT at 72 hpi. The KEGG cell cycle pathway was visualized using Pathview, with differential gene expression data from the R77Q vs. WT comparison at 72 hpi overlaid onto the pathway map. Genes significantly upregulated in R77Q relative to WT are shown in red, while significantly downregulated genes are shown in blue. Uncolored nodes indicate genes that were not differentially expressed. The predominance of red nodes reflects broad upregulation of cell cycle–associated genes in R77Q compared to WT infection. [file Image1.tif]

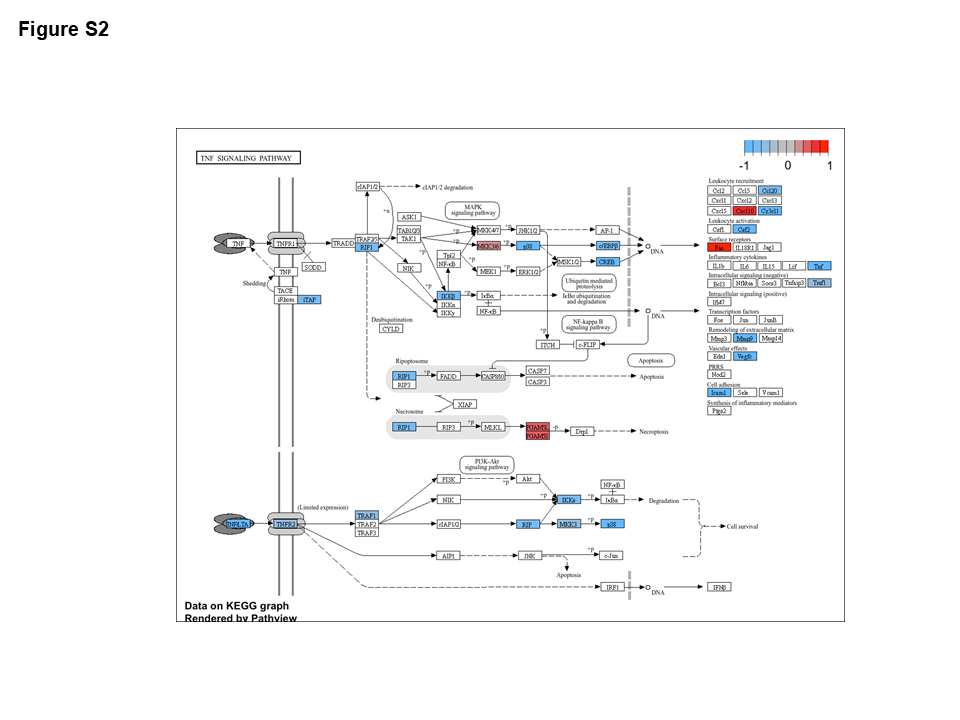

Supplement: Supplementary Figure 2 — TNF signaling pathway analysis in R77Q vs. WT at 72 hpi. The KEGG TNF signaling pathway was visualized using Pathview, with differential gene expression data from the R77Q vs. WT comparison at 72 hpi overlaid onto the pathway map. Genes significantly upregulated in R77Q relative to WT are shown in red, whereas significantly downregulated genes are shown in blue. Uncolored nodes represent genes that were not differentially expressed. The predominance of blue nodes indicates broad downregulation of TNF signaling components in R77Q compared to WT infection. [file Image2.tif]
