# Supplementary material for: The HIV-1 Vpr R77Q mutant alters host apoptotic gene regulation in CD4+ T cells
Source: Front Cell Infect Microbiol. 2026 Jun 29;16:1830094. doi: 10.3389/fcimb.2026.1830094 (PMC13357995; doi:10.3389/fcimb.2026.1830094)
Supplement: Supplementary Table 1 — Top 10 most significant DEGs filtered in the R77Q vs. WT comparison. [file Table1.pdf]

Supplementary table S1: Top 10 most significant DEGs filtered in the R77Q vs. WT comparison

Gene name	Log2FC	FDR	General Related Function
**DUSP5**	-2.01	1.87E-10	Apoptosis
**ICAM1**	-1.18	4.62E-10	Inflammation
**UNG**	1.08	9.97E-09	Inflammation
**CDCP1**	-2.12	2.48E-08	DNA damage repair (BER)
**PLD6**	1.07	4.24E-08	Mitochondrial fusion
**ULBP2**	-1.16	1.22E-07	Inflammation
**ARG2**	-1.25	1.22E-07	Mitochondrial protein
**SERPINB9**	-1.41	1.70E-07	Apoptosis
**TREML2**	-1.23	1.86E-07	Inflammation
**SUPT16H**	0.714	2.21E-07	DNA replication and repair
